# Hepatocyte Growth Factor from a Clinical Perspective: A Pancreatic Cancer Challenge

**DOI:** 10.3390/cancers7030861

**Published:** 2015-09-03

**Authors:** Wasia Rizwani, Amanda E. Allen, Jose G. Trevino

**Affiliations:** 1 Department of Biochemistry, Osmania University, Hyderabad, Telangana 500007, India; E-Mail: wasia.rizwani@gmail.com; 2 Department of Surgery, University of Florida, 1600 SW Archer Rd, Rm 6175, P.O. Box 100109, Gainesville, FL 32610, USA; E-Mail: amanda.allen@ufl.edu

**Keywords:** pancreatic cancer, HGF, desmoplasia, hypoxia, acidosis, chemotherapy

## Abstract

Pancreatic cancer is the fourth leading cause of cancer-related deaths in the United States and incidence rates are rising. Both detection and treatment options for pancreatic cancer are limited, providing a less than 5% five-year survival advantage. The need for new biomarkers for early detection and treatment of pancreatic cancer demands the efficient translation of bench knowledge to provide clinical benefit. One source of therapeutic resistance is the pancreatic tumor microenvironment, which is characterized by desmoplasia and hypoxia making it less conducive to current therapies. A major factor regulating desmoplasia and subsequently promoting chemoresistance in pancreatic cancer is hepatocyte growth factor (HGF), the sole ligand for c-MET (mesenchymal-epithelial transition), an epithelial tyrosine kinase receptor. Binding of HGF to c-MET leads to receptor dimerization and autophosphorylation resulting in the activation of multiple cellular processes that support cancer progression. Inhibiting activation of c-MET in cancer cells, in combination with other approaches for reducing desmoplasia in the tumor microenvironment, might significantly improve the success of chemotherapy. Therefore, HGF makes a potent novel target for developing therapeutic strategies in combination with existing drugs for treating pancreatic adenocarcinoma. This review provides a comprehensive analysis of HGF and its promising potential as a chemotherapeutic target for pancreatic cancer.

## 1. Introduction

Pancreatic cancer remains the fourth leading cause of cancer related deaths in the USA [1] with a five-year survival rate of only 7%. In 2014, American Cancer Society figures showed 46,420 people (23,530 men and 22,890 women) received a diagnosis of pancreatic cancer and 39,590 people (20,170 men and 19,420 women) died of this disease (www.cancer.org), and these numbers show no sign of improving in the near future. Unfortunately, even while we have made strides in the management and stabilization of other solid organ tumors, the rate of pancreatic cancer diagnoses has been increasing over the past 10 years with pancreatic cancer-related deaths only second to lung cancer (www.cancer.org). Treatment options are limited; surgery remains the only chance for survival. However, potentially curative surgical intervention is offered to less than 20% of patients diagnosed with pancreatic cancer, suggesting the aggressive nature of this disease upon clinical presentation. While current adjuvant therapies including chemotherapy and radiation have a modest effect on tumor growth, several recently investigated biological pathways associated with pancreatic cancer show promise as targeted therapies. This review will focus on the role of hepatocyte growth factor (HGF), also referred to as scatter factor (SF), as a therapeutic target in the treatment of pancreatic cancer.

## 2. HGF-the Good, the Bad and the Ugly

While twenty-five years have passed since HGF was discovered [2,3,4], its mitogenic role is still being explored. HGF is produced by stromal cells of mesenchymal origin and stimulates multiple cellular functions in various organs via tyrosine phosphorylation of its receptor, c-MET (Mesenchymal-Epithelial Transition) [5]. The functions of HGF in organ morphogenesis, regeneration, repair, and cellular diseases have been comprehensively reviewed by Nakamura *et al* [6]. *In utero*, HGF-neutralization or c-MET gene silencing can lead to organ hypoplasia in fetal stages of many organs, indicating that HGF signals are essential for organ development [7]. While endogenous HGF is essential for self-repair of injured visceral organs, lungs and other tissues [8,9,10], HGF also exerts protective effects on epithelial and non-epithelial organs such as the heart and the brain by evading apoptosis and inflammation [11,12]. During exocrine pancreatic morphogenesis, HGF levels remain significantly increased and blocking HGF ligand activity resulted in accelerated tissue destruction in a murine model [13]. Additionally, insufficient production of HGF after organ development can lead to organ failure [6]. The emerging picture in entirety is that the physiologic balance of HGF secretion is necessary for homeostasis, and HGF supplementation may in some instances be therapeutic for pathological conditions.

Unfortunately, a variety of human malignancies can take advantage of the HGF ligand/c-Met pathway activation as a mechanism for tumor promotion. Specifically, the hyperactivation of this pathway through overexpression of the HGF ligand by cells from the tumor microenvironment and overexpression of the c-Met receptor on the cancer cell lead to significant upregulation of a variety of tumor promoting signaling pathways [14,15]. This phenomenon was demonstrated in pre-clinical studies, where patient-derived stromal tissues expressing HGF correlated with enhanced invasion of pancreatic cancer cells with only high expression of c-Met [16]. Additionally, HGF exerted a resistance to anoikis on pancreatic cancer cells by phosphorylating Akt and also promoting invasion and metastasis [17]. Current work from our group also demonstrates a similar effect when silencing of the c-Met receptor in pancreatic cancer cells co-cultured with human-derived pancreatic stromal elements expressing HGF ligand resulted in abrogation of proliferation, invasion, and metastasis These results established a relationship between stromal and cancerous elements with respect to the HGF/c-MET signaling pathway in pancreatic cancer pre-clinical studies. We propose that an in-depth analysis of the HGF/c-MET signaling system in pancreatic cancer could bridge the gap between basic biology and translational medicine.

**Figure 1:**
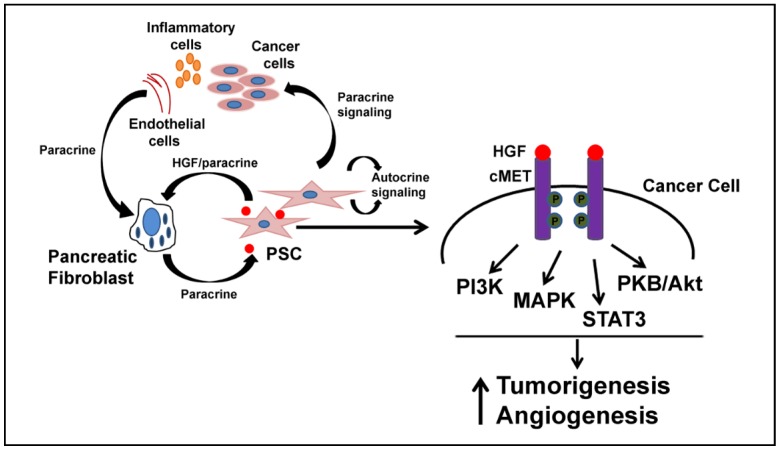
Role of pancreatic stellate cells (PSC) in desmoplasia. In its quiescent state, the pancreatic stellate cell contains vitamin A-containing lipid droplets and serves as a reservoir for Vitamin A in the normal pancreas. Its activation from a quiescent to an activated state, including changes to its proliferation rate, morphology, and sensitivity to mitogenic factors, are all primary features of pancreatic ductal adenocarcinoma (PDAC). While the activation process is not yet fully understood, hepatocyte growth factor (HGF) expression is one of the many signaling events leading to stellate cell activation. Following activation, the PSC loses its lipid droplets, undergoes morphological changes, and upregulates alpha smooth muscle actin and collagen. Intercellular signaling originating from multiple cell types, including tumor cells, endothelial cells and immune cells, contribute to this increased activation and proliferation of PSCs in the desmoplastic reaction. PSC-derived HGF can in turn activate cancer cells to promote tumorigenesis and endothelial cells to promote angiogenesis in tumors. HGF-Hepatocyte growth factor, MET-mesenchymal-epithelial transition.

## 3. HGF and Desmoplasia

Pancreatic cancer is pathologically characterized by a strong desmoplastic reaction involving up to 90% of the tumor volume, suggesting a vital role in pancreatic tumor growth [18,19,20]. Not surprisingly, chronic pancreatitis, a risk factor for pancreatic ductal adenocarcinoma (PDAC), also shows marked desmoplasia further suggesting the important role of the pancreatic microenvironment as a tumor-promoting and supporting factor during inflammatory changes of the pancreas [21,22,23]. Pancreatic tumors undergo desmoplasia through proliferation of activated fibroblasts (referred to as myofibroblasts or pancreatic stellate cells), infiltration by immune cells and deposition of multiple ECM (extracellular matrix) components with a concomitant increase in interstitial fluid pressure [23] (Figure 1). These ECM components can be cellular and non-cellular and are regulated by many intrinsic and extrinsic biological pathways [24]. Cancer cells secrete TGF-β that induces quiescent fibroblasts to adopt a myofibroblastic phenotype by increasing the expression of α-smooth muscle actin, collagen, and fibronectin [25,26,27]. In turn, the myofibroblasts can produce factors like HGF that can promote malignant changes in epithelial cells through a variety of signaling mechanisms [25,28,29]. The signaling pathways that initiate tumorigenesis and subsequently a desmoplastic reaction need to be scrutinized as it is still uncertain whether early changes in the microenvironment promote malignant changes, or if epithelial cells with malignant transformation promote a supportive desmoplastic microenvironment. What is understood is that interactions between the epithelial cells and the stromal fibroblasts govern the normal functioning of an organ/tissue. With this in mind, it is plausible that deregulation of this balance might affect the “normal” function of the native tissue and/or surrounding tissue by autocrine or paracrine signaling leading to tumor formation.

Useful insights may be gained from examining what is known of related pathways in another cancer type. In colon cancer, it has been reported that both epithelial and stromal factors are responsible for intestinal transformation and progression to tumorigenesis. Initiation of colorectal cancer is associated with loss of paracrine hormones guanylin and uroguanylin, the endogenous ligands for the tumor suppressor guanylyl cyclase C (GUCY2C), which regulates epithelial cell dynamics and balance in proliferation, metabolism and differentiation at the crypt-surface axis of the intestine [30,31,32]. Whereas the presence of GUCY2C inhibits desmoplastic reaction in colon cancer cells, elimination of GUCY2C induces desmoplasia by promoting Akt-dependent TGF-β secretion, activation of fibroblasts, and Smad3 phosphorylation. These events lead to HGF secretion by fibroblasts, which in turn drive colon cancer cell proliferation through c-MET-dependent signaling [33]. Studies in pancreatic cancer revealed that inhibiting TGF-β production by cancer cells facilitates fibroblast-derived HGF-induced tumor cell invasion [34]. Treatment of pancreatic cancer cells with HGF stimulated cell growth by enhancing TGF-α level [35]. The importance of rising HGF in pancreatic cancer *in vivo* is suggested by studies that have demonstrated the rise of HGF serum levels as the disease advances [36,37]. However, its precise role in pancreatic desmoplasia is not fully understood. Upregulation of various growth factors like TGF-β [38], platelet-derived growth factor (PDGF, [39]) and pro-inflammatory cytokines like tumor necrosis factor α, and Interleukins-1 and 6 [40] have demonstrated promotion of proliferation and migration of pancreatic stellate cells, which support a strong desmoplastic reaction. While their association with HGF has not been fully elucidated in pancreatic cancer, what is understood is that the expression of IL-1α by pancreatic cancer cell lines promotes HGF production in stromal cells to facilitate tumor-promoting properties [41]. In other tissues such as human mesenchymal stem cells, TNF-α (which supports stromal desmoplastic changes) increases the mRNA levels of HGF and also of secretory HGF in conditioned media via p38MAPK and PI3K/AKT pathways to enhance migration and autocrine production of HGF [42]. Similarly, IL-1α and IL-1β stimulation of cultured corneal fibroblasts (keratocytes) upregulates HGF and keratinocyte growth factor (KGF), thereby facilitating healing of the wounded corneal epithelial cells by modulating proliferation, motility and differentiation [43]. In colonic epithelial cells, both HGF and KGF are enhanced in IL-1 stimulated production of IL-8 during mucosal inflammations, and IL-8 expression, although promoted as an angiogenic factor in pancreatic tumors, might be playing a significant role in supporting this tumor microenvironment [44,45,46,47,48]. So, although there is a suggested link between HGF and multiple cytokines that promote a desmoplastic reaction in the tumor microenvironment, the role of HGF through similar pathways in pancreatic cancer is a topic for future research endeavors.

## 4. HGF and Hypoxia

Desmoplasia and hypoxia are intertwined events leading to aggressive PDAC. Desmoplasia promotes a hypoxic environment in pancreatic cancers and limits cancer drug delivery due to decreased blood perfusion. Tumor hypoxia activates hypoxia-inducible factor-1α (HIF-1α) that in turn activates a number of signaling pathways leading to stronger desmoplastic reaction. HIF-dependent pathways activate MET in pancreatic tumor cells, while stroma-secreted HGF facilitates cell motility from the hypoxic regions of the tumors to the oxygen-rich distant organs [49,50]. One of the prominent pathways that HIF-1α activates in pancreatic cancer cells is Sonic hedgehog signaling (SHH) that is responsible for increased fibrous tissue deposition. A positive loop of increased SHH ligand and HIF-1α production occurs that will continue to decrease blood flow and increase hypoxia [51]. It is well established that hypoxic tumors mediate angiogenesis, invasion, and malignancy in cultures [52,53,54] as well as in mouse models [55]. During hypoxia-induced angiogenesis, HGF facilitates cancer cell-endothelial cell contact through FAK (focal adhesion kinase) phosphorylation [56] and decreases endothelial occludin, a primary protein in endothelial tight junctions [57]. As a result of the tight junction morphological change, HGF increases permeability between vascular endothelial cells and promotes movement across an endothelial cell barrier into adjacent tissues. HGF-induced FAK phosphorylation simultaneously upregulates many matrix metalloproteinases like MMP-1, -9, and -14, through activation of the transcription factor Ets, in cancers of the gallbladder, prostate, and liver thereby facilitating cancer invasion [58,59,60]. Studies using pancreatic PK8 and fibroblast MRC5 cells demonstrated that hypoxia induces HGF production in culture media of MRC5 cells. PK8 cells exposed to conditioned media collected from HGF-expressing MRC5 cells showed a much higher increase in MMP-2, -7, MT1-MMP and c-MET levels, as well as a concomitant increase in c-MET phosphorylation leading to enhanced migration and invasion through the basement membrane [61].

Although PDACs are not highly vascularized tumors, several lines of evidence reveal a positive correlation between blood vessel density, tumor VEGF-A levels, and disease progression [62,63], highlighting a potential role in cancer progression. Pancreatic cancer cells themselves secrete a number of mitogenic factors with angiogenic properties, such as EGF, TGF-α, HGF, FGF-1, 2, and 5, and PDGF-β [64,65]. HGF by itself is a very potent angiogenic factor that promotes endothelial cell motility and growth via the MET receptor [52]. It has been shown in murine colon carcinoma CT6 cells that intracellular HGF increases VEGF-A mRNA levels through PI3K/Akt, MAPK, and STAT3 pathways, more so under hypoxic conditions [66]. These data reiterate the fact that a tumor-stroma interactive loop exists and facilitates tumor growth and metastasis. What remains to be explored is how similar these mitogenic and angiogenic signaling pathways work in a pancreatic environment using an orthotopic animal model of pancreatic cancer.

## 5. HGF and Acidic Environment

Physical properties of the tumor microenvironment also influence tumor progression [67]. Both hypoxia [68] and acidosis [69] can be cytotoxic, but tumor cells adapt to these stressful conditions, avoid apoptosis, [70] and survive to spread to distant sites [71]. Normal cells undergo a low rate of glycolysis followed by oxidation of pyruvate, [72] while cancer cells undergo the “Warburg effect” to produce energy by a high rate of glycolysis followed by lactic acid fermentation in the cytosol [73]. PDAC cell line PANC1 revealed alteration in metabolic pathways similar to the Warburg effect when compared to the proteome of normal pancreatic duct cells [74] via abnormal metabolism of glutamine. This suggests that glutamine is largely consumed as a nitrogen donor in nucleotide and amino acid biosynthesis, further supporting current data that demonstrates pancreatic cancer cell growth is stimulated by a glutamine-associated KRAS dependent pathway [75]. Both extracellular pH (pH_e_) and cytoplasmic pH (pH_i_) are affected due to enhanced metabolism and glycolysis by tumor cells. The pH of the cells plays a regulatory role in many cellular processes like cell cycle, motility, membrane potential, intracellular homeostasis, and eventually malignant transformation [76,77,78]. Sodium-proton exchangers (NHEs) and other transporters maintain the pH_i_ and expel protons outside the cells creating an acidic pH_e_ [79] that aggravates invasive properties of transformed cells [71,80,81]. Among growth factors, HGF is known to activate NHE directly and lowers the pH_e_ thereby inducing cathepsin-mediated trafficking of lysosomes to the cell periphery [82]. This process leads to enhanced ECM proteolysis, migration, and invasion. A study by Steffan *et al.* [83] in prostate cancer cells revealed that HGF induces lysosome trafficking to the cell periphery by phosphorylating MET receptor and activating kinase cascades like PI3K and Rho A GTPases. HGF treatment also resulted in increased microtubule accumulation at the cell surface protrusions coinciding with the lysosomes, NHE activity, and cathepsin B secretion; all these processes eventually lead to enhanced invasion by prostate tumor cells [83]. Again, such explicit evidence as is seen with prostate cancer is lacking in pancreatic tumor research, and whether the acidic environment can activate the HGF-c-MET pathway needs further investigation.

## 6. HGF and Inflammation

Inflammatory cells are an integral part of the desmoplastic reaction found in pancreatic cancer and contribute to the advancement of this disease. Chronic pancreatitis, or chronic inflammation of the pancreas, is a known risk factor for developing PDAC [84]. Inflammatory signals are implicated in both tumor initiation and tumor progression. Leukocytic infiltrates in PDAC are largely immunosuppressive and associated with reduced survival in humans [85,86]. Furthermore, pro-inflammatory markers, such as IL-6, IL-8, IL-10, and IL-1 receptor antagonist, are elevated in the serum of patients with pancreatic cancer, with IL-6 specifically being an indicator of poor prognosis [87]. IL-6 functions in pancreatic tumor progression by activating the signal transducer and activator of transcription 3 (STAT3), as well as NF-κB in macrophages [88]. Inflammatory mediators also characterize pancreatic neoplasms that account for up to 10% of pancreatic malignancies and up to 30% of pancreatic resections. HGF was highly expressed in pancreatic inflammatory cystic fluid (PIC) and implicated in distinguishing PIC from mild branch duct intraductal papillary mucinous neoplasms [89]. HGF expression can serve as a biomarker in such cases and its presence could be a possible early indicator of pancreatic malignancy.

## 7. HGF/c-MET as a Target

Desmoplasia is a contributing factor to chemoresistance, which remains a principal challenge in pancreatic cancer treatment. Pancreatic stellate cells enhance fibrosis, partly through HGF production, leading to sequestration of chemotherapeutic agents in the stromal compartment, impairing successful drug delivery to cancer cells [90,91,92]. Further studies to find novel therapeutic agents targeting the HGF-c-MET signaling axis will better help us understand the implications of HGF-c-MET signaling in malignancy. Existing agents can be explored based on their target components, whether it is HGF or c-MET.

It is well known that c-MET is deregulated in many human cancers including pancreatic cancer [93,94,95,96] and can be activated by genetic mutations, gene amplifications, protein overexpression, or a ligand-dependent autocrine/paracrine signaling loop [97,98,99]. HGF is the only known ligand for c-MET [94] and c-MET has clearly been associated with aggressive disease, poor prognosis, worse clinical outcomes, and chemoresistance in many cancers. Many ongoing clinical trials in phase I, II, and III, outlined in Table 1 below, employ MET kinase inhibitors or MET monoclonal antibodies (MAb), (Onartuzumab from Genentech) as a potent chemotherapeutic approach to tackle different cancers (www.vai.org/metclinicaltrials; [100]). Most advanced in clinical development among the c-MET targeted therapies in trial studies, Tivantinib (ARQ 197), a non-adenosine triphosphate-competitive c-MET inhibitor, is in phase III development for various malignancies [101]. Specifically for pancreatic cancer, it is under a randomized phase 2 study of ARQ 197 *versus* gemcitabine in treatment-naive patients with unresectable locally advanced or metastatic pancreatic adenocarcinoma [102]). Recent evidence with another c-MET inhibitor, INC280, demonstrated reduced motility of pancreatic cancer cells with a 30% lymph node involvement in the treatment group when compared to 60% involvement in the control group, suggesting potential suppression of metastasis [103]. Other notable results from the study include reduced motility of endothelial cells, impaired tumor growth in response to HGF, and improved gemcitabine efficacy when used in combination with the frequently prescribed nucleoside inhibitor [103].

**Table 1:** Current HGF/cMET Target Therapies in Phase II/III clinical trials. To date, multiple therapies targeting the HGF/cMET pathway are showing promising results in median progression free survival when applied to a diverse range of neoplastic pathologies supporting further exploration of HGF as a potent medical therapy avenue yet to be fully exploited.

Category	Drug Name	Trial Phase	Target Neoplasm	Median Progression Free Survival	Side Effects	Conclusions	Source
HGF/SF Mab	AMG102 (Rilotumumab) +Bevacizumab	II	Renal cell carcinoma	3.7 months at 10 mg/kg and 2 months at 20 mg/kg	Edema (45.9%)	AMG102 is tolerated, but not definitively growth inhibitory	Schoffski *et al*. [104]
					Fatigue (37.7%)		
					Nausea(27.9%)		
HGF/SF Mab	AMG 102 (Rilotumumab) *vs.* AMG 102 after previous Bevacizumab Therapy	II	Recurrent Glioblastoma	[AMG102 only] 4.1 weeks *vs.* [previous Bevacizumab] 4.3 weeks	Fatigue (38%),	AMG 102 monotherapy not associated with statistically significant anti-tumor activity	Wen *et al*. [105]
					Headache (33%)		
					Peripheral Edema (23%).		
HGF/SF Mab	AMG 102 (Rilotumumab) plus mitoxantrone and prednisone	II	Castration Resistant Prostate Cancer	3.0 months [AMG 102] *vs.* 2.9 months [control]	Pulmonary Embolism (6%)	Addition of AMG 102 showed no efficacy improvements	Ryan *et al.* [106]
					Fatigue (3%)		
Met Kinase Inhibitor	Tivantinib plus Erlotinib *versus* Placebo plus Erlotinib	III	Nonsquamous, Non-Small-Cell Lung Cancer	3.8 months [Erlotinib + Tivantinib] *vs.* 2.3 months for Erlotinib+Placebo	Rash	Addition of Tivantinib showed a significant delay in metastasis when compared to Erlotinib alone	Scagliotti *et al.* [107]
					Diarrhea		
					Fatigue		
					Vomiting		
					Dyspnea		
Met Kinase Inhibitor	Tivantinib *vs.* Placebo	II	Hepatocellular Carcinoma	1–6 months [Titantivib] *vs.* 1–4 months [Placebo]	Neutropenia (14%)	Beneficial second line treatment for c-MET-high advanced HCC.	Santoro *et al.* [108]
					Anemia (11%)		
Met Kinase Inhibitor	Tivantinib	II	Microphthalmia transcription factor (MITF)-associated (MiT) tumors	3.6 months [overall] *vs.* 5.5 months [ASPS] *vs.* 1.9 months [CCS and tRCC]	Anemia (4%)	Safe and tolerable at doses of 360mg BID, with moderate antitumor response	Wagner *et al.* [109]
					Neutropenia (4%).		
					Thrombocytopenia		
					Deep vein thrombosis (6.4%)		
Met Kinase Inhibitor	PF-02341066 (Crizotinib) *vs.* Pemetrexed or Docetaxel	III	ALK+ Non-Small Cell Lung Cancer	7.7 months [crizotinib] *vs.* 3.0 months [control]	Visual disorder	Crizotinib is superior to standard chemotherapy in terms of progression free survival, symptomology, and quality of life	Shaw *et al.* [110]
					GI SE		
					Elevated liver aminotransferase levels		
Met Kinase Inhibitor	Cabozantinib	III	Medullary Thyroid Carcinoma	11.2 months [cabozantinib] *vs.* 4.0 months [placebo]	Diarrhea	Cabozantinib resulted in statistically significant increased progression free survival length of time.	Elisei *et al.* [111]
					Palmar-plantar erythrodysesthesia Decreased weight and appetite Nausea		
					Fatigue		
Met Kinase Inhibitor	Foretinib	II	Papillary Renal Cell Carcinoma	9.3 months [Foretinib] *vs.* 1.3 months [Sunitinib]	Fatigue, Hypertension, Gastrointestinal toxicities	Foretinib demonstrated a high response rate in cancers with known germline MET mutations	Choueiri *et al.* [112]
					Pulmonary Emboli.		
Met Kinase Inhibitor	Foretinib	II	Gastric Cancer	1.7 months *vs.* [no comparison]	Hypertension (35%)	Foretinib is an insufficient monotherapy in the treatment of gastric cancer	Shah *et al.* [113]
					Elevated Aspartate Aminotransferase (23%)		

In addition to c-MET itself, the ligand HGF is an obvious therapeutic target considering its significant role in promoting tumorigenesis in cases exhibiting MET mutation [114]. Clinical trials with HGF/SF monoclonal antibody (MAb) in combination with other chemotherapeutic drugs are currently in progress. HGF/SF Mab therapies under different phases of ongoing clinical trials for various cancers include Rilotumumab from Amgen, Ficlatuzumab from AVEO pharmaceuticals and HuL2G7 from Millennium pharmaceuticals (www.vai.org/metclinicaltrials; [100]). Another promising therapy, NK4, an intra-molecular fragment of HGF, has been shown to possess anti-growth, anti-metastasis, anti-angiogenic abilities in addition to showing reduction in ascites, thereby prolonging survival in an orthotopic mouse model of pancreatic cancer [115,116].

**Figure 2:**
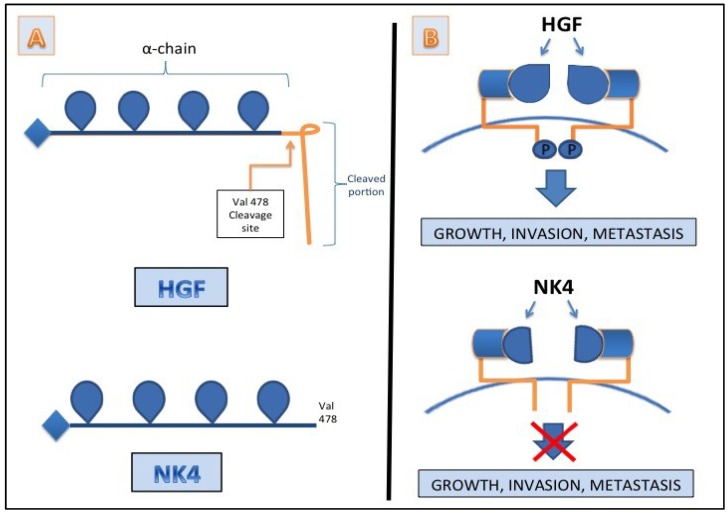
The antagonistic role of NK4 in relationship to HGF. (**A**) The intramolecular fragment of HGF: NK4; (**B**) NK4 acting as a direct antagonist of HGF with receptor binding capability while simultaneously lacking C terminus amino acids necessary for the signal transduction.

It is composed of an N-terminal hairpin domain and 4-kringle domains (K1–K4) of HGF α-chain [117,118,119] and lacks 16 amino acids from the C-terminus of HGF, and has been shown to bind MET without activating the receptor signal transduction, as shown in Figure 2. The benefits of using NK4 as an HGF antagonist have been comprehensively discussed in the review by Mizuno *et al.* [120]. With such compelling evidence, NK4 is a plausible option to target HGF along with c-MET and other drugs currently in use for controlling pancreatic cancer.

While present knowledge of HGF/c-MET has supported clinical trials targeting various aspects of the biological mechanism, more mature studies addressing side effects and clinical outcomes specific to pancreatic cancer are lacking. Combining promising c-MET and HGF antagonists along with strategic utilization of current treatment regimens leave the pancreatic cancer community with an expectant outlook for the future of medical intervention.

## 8. Conclusions

Pancreatic cancer remains a major unsolved problem, with inadequate medical therapies. HGF and its receptor counterpart are emerging as an attractive target to be exploited not only in early detection of pancreatic cancer, but also as a target for chemotherapy. Further study of these possibilities is motivated by increasing appreciation of the importance of associated pathways in other cancers and model systems. Available small-molecule and antibody-based therapeutics targeting these pathways should be investigated for utility in pancreatic cancer specific studies, and related biomarkers investigated for potential use as diagnostic tools. Additionally, focus beyond the molecular pathway’s direct consequences to include the tumor microenvironment and downstream effects on tumor behavior continue to keep HGF at the forefront of research focused on neoplastic invasion.
